# Risk factors and knowledge associated with high unintended pregnancy rates and low family planning use among pregnant women in Papua New Guinea

**DOI:** 10.1038/s41598-020-79103-6

**Published:** 2021-01-13

**Authors:** Elizabeth Peach, Christopher Morgan, Michelle J. L. Scoullar, Freya J. I. Fowkes, Elissa Kennedy, Pele Melepia, Primrose Homiehombo, Lucy Au, Stanley Luchters, Alexandra J. Umbers, Andrew Vallely, Lisa M. Vallely, Angela Kelly-Hanku, Leanne J. Robinson, Brendan S. Crabb, Arthur Elijah, Peter M. Siba, William Pomat, James G. Beeson

**Affiliations:** 1 Burnet Institute, 85 Commercial Rd, Melbourne, VIC 3004 Australia; 2 Burnet Institute, Kokopo, East New Britain Papua New Guinea; 3 Jhpiego, A Johns Hopkins Affiliate, 1615 Thames St, Baltimore, USA; 4 Department of Medicine and Melbourne School of Population and Global Health, University of Melbourne, Melbourne, VIC Australia; 5 Central Clinical School and School of Public Health and Preventive Medicine, Monash University, Melbourne, VIC Australia; 6 Murdoch Children’s Research Institute, Melbourne, VIC Australia; 7 Department of Population Health, Aga Khan University, Nairobi, Kenya; 8 Department of Public Health and Primary Care, Ghent University, Ghent, Belgium; 9 Papua New Guinea Institute of Medical Research, Goroka, Papua New Guinea; 10 The Kirby Institute, University of New South Wales, Sydney, NSW Australia; 11 Port Moresby General Hospital, and the University of Papua New Guinea, Port Moresby, Papua New Guinea

**Keywords:** Disease prevention, Public health, Epidemiology, Risk factors

## Abstract

Unintended pregnancy is a major driver of poor maternal and child health in resource-limited settings. Data on pregnancy intention and use of family planning (FP) is scarce in Papua New Guinea (PNG), but are needed to inform public health strategies to improve FP accessibility and uptake. Data from a facility-based cross-sectional sample of 699 pregnant women assessed prevalence and predictors of unintended pregnancy and modern FP use among pregnant women in East New Britain Province, PNG. More than half (55%) the women reported their pregnancy as unintended. Few (18%) reported ever having used a modern FP method, and knowledge of different methods was low. Being single, separated or divorced (AOR 9.66; 95% CI 3.27–28.54), educated to a tertiary or vocational level (AOR 1.78 CI 1.15–2.73), and gravidity > 1 (AOR 1.43 for each additional pregnancy CI 1.29–1.59) were associated with unintended pregnancy; being accompanied by a male partner to ANC was associated with a reduced unintended pregnancy (0.46 CI 0.30–0.73). Factors associated with modern FP use included male partner involvement (AOR 2.26 CI 1.39–3.67) and gravidity > 1 (AOR 1.54 for each additional pregnancy CI 1.36–1.74). FP use also varied by the facility women attended. Findings highlight an urgent need for targeted interventions to improve FP knowledge, uptake and access, and male partner involvement, to reduce unintended pregnancies and their complications.

## Introduction

Unintended pregnancies expose women to obstetric risks arising from undesired fertility, unsafe abortions, inadequate birth spacing, and pregnancies in high risk groups^1–5^. Family planning (FP) using modern contraceptives has reduced the number of maternal deaths globally by 40% over the past two decades^6^. In 2012, 220 million women were estimated to still have an unmet need for FP. If all women who wanted to avoid pregnancy were able to access effective FP, an estimated 54 million unintended pregnancies, 21 million unplanned births, 16 million unsafe abortions, 1.1 million infant deaths and 118,000 maternal deaths would have been prevented^3^. This represents a 30% reduction in maternal deaths globally^6^. Additionally, an ability to adequately space births and achieve desired family size enables women to participate in the workforce and to achieve higher levels of education for themselves and their children^2,7^.

At an estimated 594 per 100,000 live births, in 2013 Papua New Guinea (PNG) has one of the highest maternal mortality ratios (MMR) in the world; it was one of only 16 countries globally to have a persistently high MMR between 500 and 1000, between 1990 and 2013^8^. Evidence suggests that prevalence of unmet need for FP among women of childbearing age who were married or in union did not improve in PNG in the two decades from 1990 to 2010, making it one of 42 countries globally with an unmet need greater than 25%^9^. In 2015, an estimated 317,000 women of childbearing age in PNG had an unmet need for FP, and this is projected to increase to 337,000 by 2030^10^. Fulfilling unmet needs for FP may prevent an estimated nearly half (47.4%) of all maternal deaths in PNG^11^. The Government of PNG and its donor partners have intensified efforts to reduce maternal mortality primarily through increased access to and uptake of modern methods of FP^12,13^. Accordingly, increasing population coverage of FP is a target of PNG’s National Health Plan 2011–2020^14^ and an important element of the country’s contribution to global Sustainable Development Goals (SDGs)^15^. However, additional data on factors associated with unintended pregnancy and FP use are needed to help achieve these goals.

There are significant geographic, social and cultural barriers and health system constraints to delivering modern FP and maternal and child health (MCH) services in PNG^16–20^, and data on unintended pregnancy and FP use among women of childbearing age to inform evidence-based public health strategies and interventions is limited. PNG’s latest published Demographic and Health Survey (DHS) from 2016 estimates that women have on average 1.2 more children than they desire^21^. Results from a cross-sectional survey of pregnant women attending multiple antenatal clinics (ANC) at Port Moresby General Hospital in PNG’s capital city suggest that around half of pregnancies in this population are unintended ^22^. PNG’s DHS also found that over 80% of women and men aged 15–49 years could recall a modern method of FP, but only 37% reported ever having used a modern method of FP^21^. However, knowledge of factors associated with unintended pregnancy and FP use is limited. This paper reports prevalence and predictors of unintended pregnancy and FP use from a cross-sectional sample of pregnant women attending their first ANC visit in East New Britain (ENB) Province, PNG. This study aimed to generate findings to inform public health strategies to facilitate improved access to and uptake of a range of FP options and reduce unintended pregnancies.

## Methods

This study included analysis of cross-sectional baseline data from a larger prospective observational cohort study of pregnancy and childbirth undertaken in ENB Province, by a multi-partner research program led by the Burnet Institute with the PNG Institute of Medical Research, the ENB Provincial Health Office (now Provincial Health Authority) and the Kirby Institute of the University of New South Wales, Sydney. Input from relevant national and local stakeholders, including policy-makers, medical specialists, and healthcare workers at all levels, was sought during 2013 and 2014 to inform the design of the study. This paper reports on interview data relevant to FP collected at the enrolment contact point at the first ANC visit.

### Setting

ENB Province is located in the Islands Region of PNG. According to the 2011 census, ENB had a population of 328,369 and a population growth rate of 3.6% between 2000 and 2011^23^. The province is predominantly rural, reflecting PNG’s national profile wherein 87% of the PNG population reside in rural areas^24^, with two small urban centres (populations estimated at close to 32,000 (Kokopo) and 5000 (Rabaul)^23^). Pregnant women of any gravidity attending their first ANC visit were recruited from five healthcare facilities located in three of the four districts in ENB (Gazelle, Kokopo and Rabaul) where 78% of the provincial population resides^23^. They comprise a mix of two government and three church-run facilities and are the busiest providers of reproductive health services in these adjoining districts, accounting for over 75% of antenatal services, based on information provided by the Provincial Health Office in 2014. Nonga General Hospital is the government referral hospital for the province, and is located near Rabaul township. The government-run Kerevat Rural Hospital is the most remotely located of the participating facilities and is administered by the Gazelle District Health Administration. Saint Mary’s Hospital Vunapope, and Napapar and Paparatava Health Centres are administered by Catholic Health Services, PNG, under the Gazelle District Health Administration. Vunapope is located in the town of Kokopo, the capital and largest urban centre in ENB, whereas Napapar and Paparatava are smaller, rural facilities.

### Study population

Women were enrolled between March 2015 and June 2017. A target sample size of 700 was set by the larger cohort study’s parameters needed to assess predictors of low birth weight. Recruitment aimed for a representative sample of pregnant women attending ANC who were selected randomly (by rolling dice) with spacing to ensure both early and late attendees were invited. Women had to meet the following eligibility criteria; (1) age of 16 years or older; (2) attending ANC for the first time for the current pregnancy; (3) residing within the catchment area of the healthcare facility; (4) intending to live in ENB for the subsequent 12 months; (5) agree to participate in the study. Written informed consent was obtained from all study participants.

### Data collection and measures

A questionnaire with a mix of closed and open-ended questions was drafted in English and translated into Tok Pisin language, the most widely spoken national language of PNG. It was administered using electronic handheld devices by research officers of PNG nationality, trained in clinical interview techniques, using a private location at each facility to ensure confidentiality.

Outcome measures of pregnancy intention and FP use were adapted from standard FP items in the DHS women’s questionnaire^25^, and modified to the PNG context after pre-testing (including revision to include contraceptive methods currently available). Women could report their current pregnancy as either (1) mistimed (i.e., wanting to be pregnant later, but not at this time), (2) unwanted (i.e., not wanting to be pregnant at all) or (3) wanted; a pregnancy was considered unintended if it was mistimed or unwanted^26^. Women were asked if they had ever used FP, and if so, without prompting, were asked to recall all methods (modern and/or traditional) that they had ever used. Modern and traditional methods of FP were defined using World Health Organization classifications^27^ with modern methods comprising: oral contraceptive pills, implants, injectables (Depo Provera), female sterilisation, male sterilisation, intra-uterine devices, diaphragm, emergency contraception, male and female condoms.

Exposure measures comprised of questions relating to socio-demographic characteristics, male partner involvement in ANC, pregnancy history, and knowledge of FP methods. Male partner involvement was assessed by asking women whether their husband/male partner was in attendance at ANC that day, and if not, whether he would have liked to attend. Questions relating to pregnancy history (pregnancy number and number of years since the previous pregnancy) included all previous pregnancies regardless of outcome. Similar to questions on FP use, without prompting, women were asked to recall all methods of FP of which they were aware. Open-ended questions asked women to give opinions on barriers to accessing FP services if they reported that access to FP was sometimes a problem, or if they were unsure if access was difficult. They were also asked to provide reasons for non-use of FP if they reported never having used any method of FP. Research officers selected from a list of standardised response options those that best matched the woman’s answer/s, with multiple response options allowed. If a participant’s response differed from the standardised list this was captured as a free-text entry.

### Statistical analysis

Bivariate and multivariable logistic regression explored correlates of unintended pregnancy and lifetime use of a modern method/s of FP. Variables of interest were chosen for multivariable analyses a priori and included healthcare facility of recruitment and variables cited in the literature to be associated with these outcomes of interest (marital status, indicators of socio-economic status and gravidity^21,22,28^) and variables hypothesised to have an association (male involvement in ANC and reporting difficulty accessing FP). Due to collinearity between participant educational level, male partner educational level, participant employment status, male partner employment status and monthly household expenditure, only participant educational level was included in the final multivariable model. Similarly, due to collinearity between age and gravidity, only gravidity was included in the final models.

All analyses were performed using STATA version 13.0 (StataCorp, TX, USA).

### Ethics approval and consent to participate

Approval for the study protocol was granted in PNG by the Papua New Institute of Medical Research’s Institutional Review Board (14.11), the National Department of Health Medical Research Advisory Committee (14.27), and in Australia by the Alfred Hospital Human Research Ethics Committee (348/18). Approval to conduct the study was obtained from the Provincial Executive Committee of the East New Britain Provincial Government, and the individual health centres involved. Key considerations were to ensure written informed consent using local language forms and detailed explanations, minimisation of discomfort during data collection, and assurance of confidentiality through use of non-identifiable study identifiers; there was separate, limited, controlled access to any identifying information required for follow-up. Independent contact points for complaints or adverse event reporting were publicised and maintained by the Burnet Institute and PNG Institute of Medical Research. All study participants provided written informed consent. All study procedures were performed in accordance with relevant guidelines and regulations.

## Results

### Participant characteristics

A total of 699 pregnant women (median age 26 years) participated in the study. Most women (491/699; 70%) were recruited from Catholic church-administered healthcare facilities (Vunapope, Napapar and Paparatava), and most (432/699; 62%) from rurally located facilities (Kerevat, Napapar and Paparatava). Almost all (663/697; 95%) reported being married or cohabiting with their male partner. Just over half (373/698; 53%) had completed higher than primary school education, and the majority (553/699; 79%) were not in paid employment at the time of the study. One-quarter (175/699; 25%) were in their first pregnancy, whilst a minority (65/699; 9%) were grand-multigravid women with their sixth pregnancy or greater. Half (353/694; 51%) had a husband/partner who was either present at ANC (18%) or who was not but was reported to have wanted to attend (33%; Table 1).

**Table 1:** Participant characteristics at antenatal clinics.

	n	%
**Demographics**		
Age (years)^a^		
**Median (IQR)**	26 (22–30)	
16–24	275	40
25–34	334	48
35 +	83	12
**Marital status**^**b**^		
Married or cohabiting	663	95
Single/not living with partner	28	4
Separated or divorced	6	< 1
**Highest level of education completed**^**c**^		
Primary (grade 8 or less)	325	47
High school (grade 9–10)	177	25
Secondary(grade 11–12)	50	7
Tertiary	22	3
Vocational	124	18
**Highest level of education completed by husband/partner**^**d**^		
Primary (grade 8 or less)	193	28
High school (grade 9–10)	147	21
Secondary(grade 11–12)	81	12
Tertiary	35	5
Vocational	177	25
Don’t know	63	9
**Employment status**		
Not in paid work	553	79
Employed (part-time, full-time or self-employed)	146	21
**Employment status of husband/partner**^**e**^		
Not in paid work	276	40
Employed (part-time, full-time or self-employed)	419	60
**Monthly household expenditure (PNG kina)**^**f**^		
Median (IQR)	150 (50–300)	
**Religion**^**c**^		
Catholic	345	49
United	225	32
Other	128	18
**Location of healthcare facility**		
Vunapope	184	26
Nonga	83	12
Kerevat	125	18
Napapar	158	23
Paparatava	149	21
**Pregnancy history and male involvement**		
**Gravidity**		
Median (IQR; not including current pregnancy)	2 (0–3)	
First pregnancy	177	25
Second pregnancy	145	21
Pregnancy number 2–5	312	45
6th pregnancy or greater	65	9
**Number of years since previous pregnancy**^**g**^		
Median (IQR)	2 (2–4)	
**First pregnancy**	175	26
< 2	107	16
≥ 2	396	58
**Husband/partner present at ANC**^**h**^		
No	341	49
No, but would like to come	230	33
Yes	123	18
**Family planning**		
**Unintended pregnancy**^**c**^		
No	315	45
Yes; wanted to get pregnant later	188	27
Yes; did not want to get pregnant	195	28
**Heard of any method of FP**^**i**^		
No	192	28
Yes	498	72
**Could recall at least one modern method# of FP**^**i**^		
No	281	41
Yes	409	59
**Ever used any method of FP**^**i**^		
No	464	67
Yes	226	33
**Ever used a modern method# of FP**^**i**^		
No	569	82
Yes	121	18
**Reported difficulty accessing FP**^**e**^		
No	454	65
Yes	138	20
Not sure	103	15

nb Percentages may not add to 100 due to rounding.

^#^modern methods include: oral contraceptive pills, implants, injectables, female sterilisation, male sterilisation, intra-uterine devices, diaphragm, emergency contraception, male and female condoms.

^a^Missing data for 7 participants.

^b^Missing data for 2 participants.

^c^Missing data for 1 participant.

^d^Missing data for 3 participants.

^e^Missing data for 4 participants.

^f^Missing data for 36 participants.

^g^Missing data for 21 participants.

^h^Missing data for 5 participants.

^i^Missing data for 9 participants.

### Prevalence and predictors of unintended pregnancy

There was a very high prevalence of unintended pregnancy, with over half (383/698; 55%) of the women reporting their current pregnancy as unintended (27% mistimed and 28% unwanted, Table 1).

Univariate analysis identified several factors associated with higher odds of unintended pregnancy: marital status (single, separated or divorced), higher monthly household expenditure, increasing gravidity, a more recent previous pregnancy, and older age (weak effect) (Table 2). Lower odds of unintended pregnancy were associated with male partner involvement in ANC attendance and the male partner being employed in paid work. Reported FP use or access was not associated with unintended pregnancies. Although unintended pregnancy was higher among older women and multigravida women, it was still very high among younger women and primigravid women. Almost half (132/275; 48%) of those aged 16–24 years reported their current pregnancy as unintended, increasing to 66% (54/82) among those aged 35 and older. Similarly, 44% (77/177) of primigravid women reported their current pregnancy as unintended, increasing to 77% (49/64) amongst grand-multigravid women with their sixth pregnancy or greater.

**Table 2:** Factors associated with unintended pregnancy.

	n/N^a^	Unadjusted OR (95% CI)	*p*	Adjusted OR (95% CI)^c^	*p*
Unintended pregnancy (n/N = 383/698)					
**Demographics**					
Age in years (continuous)		1.04 (1.02–1.07)	**0.002**		
**Marital status**					
Married or cohabiting	351/662	1.0		1.0	
Single, separated or divorced	30/34	6.65 (2.32–19.07)	** < 0.001**	9.66 (3.27–28.54)	** < 0.001**
**Highest level of education completed**					
Primary school or less	171/324	1.0		1.0	
High school/secondary school	121/227	1.02 (0.73–1.43)	0.903	1.29 (0.89–1.86)	0.180
Tertiary/vocational	90/146	1.44 (0.97–2.14)	0.074	1.78 (1.15–2.73)	**0.009**
**Highest level of education completed by husband/partner**					
Primary school or less	115/192	1.0			
High school/secondary school	122/228	0.77 (0.52–1.14)	0.189		
Tertiary/vocational	110/212	0.72 (0.49–1.07)	0.106		
Don’t know	33/63	0.74 (0.42–1.31)	0.295		
**Employment status**					
Not in paid work	308/552	1.0			
In paid work	75/146	0.84 (0.58–1.21)	0.339		
**Employment status of husband/partner**					
Not in paid work	163/275	1.0			
In paid work	216/419	0.73 (0.54–0.99)	**0.046**		
**Monthly household expenditure (kina)**					
Less than or equal to 50	80/167	1.0			
Greater than 50	282/495	1.44 (1.01–2.05)	**0.042**		
**Religion**					
Catholic	190/344	1.0			
Other	193/353	0.98 (0.73–1.32)	0.882		
**Location of healthcare facility**					
Vunapope	99/184	1.0		1.0	
Nonga	51/83	1.37 (0.81–2.32)	0.245	1.66 (0.94–2.94)	0.081
Kerevat	72/124	1.19 (075–1.88)	0.461	1.34 (0.82–2.19)	0.237
Napapar	79/158	0.86 (0.56–1.31)	0.483	0.99 (0.63–1.58)	0.975
Paparatava	82/149	1.05 (0.68–1.62)	0.823	1.16 (0.73–1.84)	0.536
**Pregnancy history and male involvement**					
Gravidity (continuous)		1.31 (1.20–1.44)	** < 0.001**	1.43 (1.29–1.59)	** < 0.001**
**Number of years since previous pregnancy**^**b**^					
≥ 2	223/396	1.0			
< 2	76/107	1.90 (1.20–3.02)	**0.006**		
**Husband/partner present at ANC**					
No	206/340	1.0		1.0	
No, but would like to come	121/230	0.72 (0.51–1.01)	0.059	0.75 (0.52–1.07)	0.130
Yes	54/123	0.51 (0.34–0.77)	**0.002**	0.46 (0.30–0.73)	**0.001**
**Family planning**					
**Reported difficulty accessing FP**					
No or not sure	307/557	1.0		1.0	
Yes	75/137	0.99 (0.68–1.43)	0.937	0.95 (0.64–1.43)	0.818
**Ever used a modern method of FP**					
No	306/568	1.0			
Yes	73/121	1.30 (0.87–1.94)	0.196		

^a^n/N—data show number of subjects for each parameter (n), and the total sample size (N).

^b^Restricted to multiparous women (n = 503).

^c^Multivariable analyses excluded collinear variables, as defined in “Methods”.

In multivariable analysis (which omitted collinear variables as defined in “Methods”) (Table 2), women who were single, separated or divorced had almost tenfold the odds of unintended pregnancy than women who were married or co-habiting with their male partner (AOR 9.66, 95% CI 3.27–28.54). However, the great majority of women with unintended pregnancies were women with partners. Women who were educated to a tertiary or vocational level had increased odds of unintended pregnancy compared to women who completed primary school or less (AOR 1.78, 95% CI 1.15–2.73). Gravidity was also associated: the odds of reporting the current pregnancy as unintended increased by 43% (95% CI 1.29–1.59) with each subsequent pregnancy. Importantly, women who were accompanied by their male partner to ANC had decreased odds of unintended pregnancy compared with participants who were not (AOR 0.46, 95% CI 0.30–0.73).

In a sub-analysis of multigravid women (n = 503), reporting that the current pregnancy occurred less than two years after the previous pregnancy was associated with unintended pregnancy (OR 1.90, 95% CI 1.20–3.02) (Table 2).

### Prevalence and predictors of modern family planning use

Knowledge and reported use of modern methods of FP was very low. Over half (409/690; 59%) of women knew of at least one modern method but only 18% (121/690) reported ever having used a modern method (Table 1).

In univariate analysis, older age, male partner having paid employment, higher monthly household expenditure, higher gravidity and male involvement were associated with higher modern FP use (Table 3). Reported modern FP use was particularly low among younger and primigravid women, ranging from 8% (22/270) among women aged 16–24 years to 28% (23/82) among women aged 35 years and older, and from only 2% (4/176) among primigravid women to 32% (21/65) among women with their sixth pregnancy or greater. FP use also differed by the healthcare facility where women were attending.

**Table 3:** Factors associated with ever having used a modern method of family planning.

	n/N^a^	Unadjusted OR (95% CI)	*p*	Adjusted OR (95% CI)^c^	*p*
Lifetime use of a modern method of FP (n/N = 121/690)					
**Demographics**					
Age in years (continuous)		1.10 (1.07–1.14)	** < 0.001**		
**Marital status**					
Married or cohabiting	118/655	1.0		1.0	
Single, separated or divorced	3/33	0.46 (0.14–1.52)	0.200	0.87 (0.25–3.09)	0.836
**Highest level of education completed**					
Primary school or less	52/320	1.0		1.0	
High school/secondary school	40/223	1.13 (0.72–1.77)	0.606	1.44 (0.86–2.40)	0.162
Tertiary/vocational	29/146	1.28 (0.77–2.11)	0.340	1.57 (0.89–2.77)	0.118
**Highest level of education completed by husband/partner**					
Primary school or less	42/187	1.0			
High school/secondary school	37/225	0.68 (0.42–1.11)	0.124		
Tertiary/vocational	35/212	0.68 (0.41–1.12)	0.134		
Don’t know	7/63	0.43 (0.18–1.02)	0.055		
**Employment status**					
Not in paid work	94/544	1.0			
In paid work	27/146	1.09 (0.68–1.74)	0.732		
**Employment status of husband/partner**					
Not in paid work	36/268	1.0			
In paid work	85/418	1.64 (1.08–2.51)	**0.021**		
**Monthly household expenditure (kina)**					
Less than or equal to 50	23/164	1.0			
Greater than 50	92/490	1.84 (1.22–2.77)	**0.003**		
**Religion**					
Catholic	57/340	1.0			
Other	64/349	1.11 (0.75–1.65)	0.587		
**Location of healthcare facility**					
Vunapope	32/183	1.0		1.0	
Nonga	15/83	1.04 (0.53–2.05)	0.908	1.42 (0.68–2.94)	0.347
Kerevat	43/125	2.47 (1.46–4.21)	**0.001**	2.78 (1.56–4.96)	**0.001**
Napapar	24/152	0.88 (0.50–1.58)	0.679	1.18 (0.63–2.21)	0.599
Paparatava	7/147	0.24 (0.10–0.55)	**0.001**	0.21 (0.09–0.51)	**0.001**
**Pregnancy history and male involvement**					
Gravidity (continuous)		1.41 (1.27–1.57)	** < 0.001**	1.54 (1.36–1.74)	** < 0.001**
**Number of years since previous pregnancy**^**b**^					
≥ 2	89/389	1.0			
< 2	24/107	0.97 (0.58–1.63)	0.922		
**Husband/partner present at ANC**					
No	42/335	1.0		1.0	
No, but would like to come	56/230	2.25 (1.44–3.49)	** < 0.001**	2.26 (1.39–3.67)	**0.001**
Yes	23/121	1.64 (0.94–2.86)	0.083	1.48 (0.81–2.72)	0.207
**Family planning**					
**Reported difficulty accessing FP**					
No or not sure	102/550	1.0		1.0	
Yes	19/137	0.70 (0.41–1.19)	0.188	0.74 (0.41–1.33)	0.313

^a^n/N—data show number of subjects for each parameter (n), and the total sample size (N).

^b^Restricted to multiparous women (n = 503).

^c^Multivariable analyses excluded collinear variables, as defined in “Methods”.

In multivariable analysis, compared with women who were seen at Vunapope hospital (a town-based church-administered facility), women who were seen at Paparatava health centre (a rural church-administered facility) had a 79% reduction in the odds of reporting use of modern FP (AOR 0.21, 95% CI 0.09–0.51). Women seen at Kerevat hospital (a rural government administered facility) had over twofold the odds of reporting use of modern FP (AOR 2.78, 95% CI 1.56–4.96). Male partner involvement was associated with higher FP use in the adjusted analysis: women who reported that their male partner was not at ANC but wanted to attend had increased odds of reporting ever having used a modern method of FP than women whose male partner was not present and did not want to attend (AOR 2.26, 95% CI 1.39–3.67). With each subsequent pregnancy, the odds of reporting ever having used a modern method of FP increased by 54% (95% CI 1.36–1.74) (Table 3).

In a sub-analysis of multigravid women (n = 503), the number of years since the previous pregnancy was not associated with reporting ever having used a modern method of FP (OR 0.97, 95% CI 0.58–1.63) (Table 3).

### Family planning methods

There was a relatively low prevalence of knowledge of different FP options (Table 4). Most (498/690; 72%) women could recall at least one method of FP, whether modern or traditional. A greater proportion of women could recall a modern method (409/690; 59%) than a traditional method (224/690; 32%). Modern methods most commonly recalled were injectables (285/690; 41%) and the oral contraceptive pill (177/690; 26%). The traditional method that was most commonly recalled by participants was the rhythm method (186/690; 27%). Of women who could recall a modern method of FP, 66% (268/409) could recall more than one modern method, but less than one-third (121/409; 30%) had ever used a modern method.

**Table 4:** Prevalence of knowledge of and use of family planning.

Family planning method/s	Heard of n	% women	Ever used, n	% women
**Modern methods**				
Injectable	285	41	90	13
Oral contraceptive pill	177	26	20	3
Implant	154	22	7	1
Female sterilisation	106	15	1	< 1
Male condom	77	11	10	1
Female condom	44	6	1	< 1
IUD	10	1	0	< 1
Male sterilisation	9	1	0	0
Emergency contraception	1	< 1	0	0
Any modern method	409	59	121	18
**Traditional methods**				
Rhythm	186	27	95	14
Withdrawal	16	2	11	2
Breastfeeding	2	< 1	2	< 1
Abstinence	5	< 1	0	0
Herbs, roots, leaves or bark	27	4	8	1
Any traditional method	224	32	113	16

nb denominator for calculation of proportions is 690 women who provided a response: participants could report more than one method.

There was a very low prevalence of reported lifetime use of FP. One-third (226/690; 33%) of women reported ever having used any method, whether modern or traditional. A similar proportion of women reported ever having used a modern method of FP (121/690; 18%) as ever having used a traditional method (113/690; 16%). Modern methods that were most commonly used were injectables (90/690; 13%) and the oral contraceptive pill (20/690; 3%). The rhythm method was the most common traditional method ever used (95/690; 14%) (Table 4). Of women who had ever used a modern method, only eight (N = 121; 7%) had ever used more than one type of modern method.

### Reasons for never using family planning, and barriers to access to family planning

Of 464 women who reported never having used any method of FP, just under half (n = 220) provided at least one reason for non-use. The most common reason for non-use, reported by 55% (120/220) of women, was insufficient knowledge about FP or how to access it. It was also common to report beliefs surrounding FP (personal objection, family objection, or fear of stigma from the community) as a reason for non-use (67/220; 30%). Other social or attitudinal factors, reported by 16% (36/220) of women, included wanting to be pregnant, or the absence of their male partner preventing them from being able to use FP (Table 5). Structural barriers were less commonly reported by those who had never used FP, with only 7% (16/220) reporting FP supply or accessibility as a reason for non-use. Most women (204/220; 93%) gave one reason only for non-use of FP, with only 7% (16/220) providing more than one.

**Table 5:** Reported reasons for never having used family planning.

	N	% women
**Demand-side barriers**		
Insufficient knowledge about FP or how to access FP	120	55
Fear of side effects or interference with body processes	29	13
Personal objection or for religious reasons	28	13
Husband objects or prohibits FP	5	2
Stigma	5	2
Never attempted to access FP	3	1
Doesn’t want FP	5	2
Husband away	6	3
First pregnancy so never used previously	2	< 1
Wanted to get pregnant	20	9
**Supply-side barriers**		
Healthcare facility refused to give	5	2
Poor supply	4	2
Inconvenient or difficult to access	6	3
Cost	1	< 1
Total number of responses from 220 women	239	

nb denominator for calculation of proportions is 220 women who reported that they had never used any method of family planning and who also provided at least one reason for non-use: participants could provide more than one reason.

Similarly, of 241 women who reported that access to FP was sometimes a problem, or that they were unsure if access was difficult, the most common reason given was lack of knowledge about FP or how to access it (33/241; 14%). Almost one-quarter (55/241; 23%) reported beliefs (of participant, male partner or community) relating to FP to be a barrier to access, including feeling that healthcare is not needed to obtain/practice FP, obstructive attitudes from other family or community members, fear of side effects and personal objections to the use of FP. Over one-quarter of women (62/241; 26%) reported service delivery problems to be a barrier, including cost and accessibility of transport and healthcare, health worker attitudes, and inadequate supply and provision of FP services (Table 6). A minority (25/241; 10%) of women gave more than one reason. Of these women, service delivery problems were commonly reported, with 32% (8/25) reporting both cost of transport and cost of healthcare to be a problem, and 32% (8/25) reporting cost (either of transport or healthcare) together with distance to the healthcare facility or with difficulty accessing transport to get to the facility. Many (103/241; 43%) women did not provide a reason why FP was difficult to access; of these women, most (80/103; 78%) had responded that they were unsure if FP was difficult to access.

**Table 6:** Reported reasons for difficulty accessing family planning.

	N	% women
**Demand-side barriers**		
Insufficient knowledge about FP or how to access FP	33	14
Feels that healthcare is not needed for this	11	5
Partner feels that healthcare is not needed for this	7	3
Community feels that healthcare is not needed for this	3	1
Obstructive attitudes from family	16	7
Obstructive attitudes from community	8	3
Fear of using FP services, including fear of side effects	8	3
Personal objection to using FP	2	< 1
Male partner approval required to use FP	3	1
Not comfortable with asking healthcare workers about FP	15	6
Other	2	< 1
**Supply-side barriers**		
Cost of transport too high	17	7
Cost of healthcare too high	10	4
Healthcare facility too far away or transport too difficult to access	13	5
Too difficult to get time away from work or home	4	2
FP not provided by healthcare facility, including for religious reasons	12	5
Staff at healthcare facility are not kind or sympathetic	2	< 1
Insufficient supply or long waiting time at healthcare facility	4	2
Did not state any specific barriers	103	43
Total number of responses from 241 women	273	

nb denominator for calculation of proportions is 241 women who reported that family planning can sometimes be difficult to access, or who were unsure if family planning was difficult to access: participants could provide more than one reason.

### Discussion

This is the first report to describe pregnancy intention and FP knowledge and use in PNG including pregnant women in urban and smaller rural health facilities in PNG, and identifying factors associated with unintended pregnancy and FP use. Our study population had very high prevalence of unintended pregnancy, low levels of knowledge of FP, and very low levels of reported lifetime use of modern methods of FP. Prevalence of unintended pregnancy in our study population (even accounting for methodological differences) appears higher than that found in a 2012 survey of antenatal attendees in PNG’s urban capital city^22^ and more than 10% higher than global and regional estimates^1^. Levels of knowledge and use of modern FP methods in our study population are lower than those found in PNG’s DHS^21^. They are also well below the United Nations estimated 28.7% of PNG women aged 15–49 married or in union^10^. Even multigravid women with a space of two years or more between most recent pregnancies were more likely to report the current pregnancy as unintended than those with a space of less than two years, reflecting this population’s low rates of pregnancy planning. If SDG targets to increase access to sexual and reproductive health services and reduce maternal mortality are to be met in this setting, evidence-based demand and supply-side interventions must be significantly scaled up, taking a whole-of-population approach to ensure universal access to FP.

Our data suggest that knowledge is an important constraint; while most women in our study were aware of at least one modern FP method, the most common barrier reported by participants was lack of adequate information and knowledge regarding how FP works or how to access it. Over half of participants who reported having never used any method of FP did not give a reason for this; it is plausible that this was also due in large part to lack of knowledge. Social and personal factors limiting demand for FP were also important, illustrated by the large discrepancy we found between women who knew of a modern method of FP and those who reported having ever used a modern method. Personal, male partner and community beliefs, attitudes and norms, including objections to the use of FP and fear of side effects were commonly reported by women to discourage seeking and using FP. Male partner involvement in antenatal care was strongly associated with reduced unintended pregnancy and higher use of modern FP, highlighting the potential benefits of including men in all public health strategies to increase uptake, accessibility and demand for FP. In PNG, as in many similar settings globally, decisions on birth spacing and FP use often involve male partners and other family members^16,29–31^; our data concur with the findings of others that family or community attitudes to FP can be a significant barrier to uptake^21,30^.

Information and education initiatives proven elsewhere in PNG and similar countries could address the demand-side barriers we documented; to improve knowledge, challenge myths and misconceptions, and promote uptake of FP. They can be provided by a range of personnel, including health care professionals, peer educators, teachers and trainers across a range of settings, including homes, schools, workplaces and community venues^31–34^. Mass media with extensive reach and appeal, such as in serial dramas or promotional material on television or radio^32,35,36^, could hold great potential in our setting where more than 70% of women and men of childbearing age in the Islands region reported listening to radio once or more per week^21^. Information services tailored to men and boys, through channels they feel comfortable accessing^37^, have been shown elsewhere to increase knowledge and uptake of FP across a range of settings^31,38^. Such interventions can also promote gender equity and successfully change gender-related attitudes and behaviours that restrict FP uptake^39^. Use of mobile telephone messaging, a preferred source of sexual and reproductive health information for young people in Pacific islands^34^, may also offer new options in our setting^40,41^.

Our findings also call for significant re-organisation and scale-up of the delivery of FP services, to meet current unmet need and any expansion of demand. In view of our finding of an over-reliance on less effective traditional FP methods, expanding the choice of methods readily available is important. Injectable contraception was the only method reported commonly by women in our sample who had used modern FP, with few reporting use of other methods, such as other long acting reversible contraception (LARC) or the oral contraceptive pill. Condom use was very rarely reported, possibly reflecting lack of knowledge of the dual function of condoms for FP as well as disease prevention, and/or difficulties in women’s negotiation of condom use with male partners^30^. This is of particular significance in the context of an extremely high burden of reproductive tract infections in the population under study^42^. PNG’s national FP policy^13^ stipulates access to a range of modern contraceptive options, in line with international recommendations^43,44^, and adequate clinical guidelines exist for PNG health care^45,46^. However, additional detailed operational guidance and practical strategies to translate policy into effective practice are needed^18,20^.

Increasing FP (LARC in particular) availability at the community level, as well as at all levels of the health care system, has been demonstrated to be highly cost-effective for improving maternal, neonatal and child health^6,18,47,48^ in various settings. Provision of contraceptive implants via outreach services has been found to be feasible and acceptable in two rural provinces of PNG^49^. Economic modelling suggests that investment in PNG of US $1.5–2 million annually between 2017–2020 towards scaling up supply of LARCs, implants in particular, had the potential to increase contraceptive prevalence to 50%. This would result in 100,000–200,000 fewer unintended pregnancies, 40,000–80,000 less unsafe abortions, and avoiding over 100 maternal deaths per year. This represents a cost-savings of US $2–3 million per year in direct pregnancy-related health care costs alone^18^.

Other service delivery changes suggested by our findings include better integration of FP information and services with other services where women of childbearing age are seen^20,50–54^, particularly integration of FP into postpartum and child health services^55,56^. High gravidity and short birth intervals among women in our study suggest there are missed opportunities to provide education, counselling and FP supplies at postnatal or child health visits, particularly for immunization. Use of mobile outreach services and community health workers^57,58^, innovative social marketing schemes^32,59,60^, voucher programs^61–63^, and home-based delivery of FP information and services integrated with other important MCH initiatives^33^ are all absent in our study area at present, and may present promising future solutions.

We also found differences in reported lifetime use of modern FP among the health facilities mothers were attending, which may reflect reduced accessibility to modern FP in certain church-administered services. This may be a significant limitation for families in rural areas who do not have access to a government provider, and it is important that alternative modes of FP service delivery for populations are implemented. Variations in access to FP may also reflect broader difficulties in the health system, for example in FP commodity supply chains. Improvement in facility-based services is a promising avenue for improvement in ENB Province, given that routine government monitoring data estimate that over 80% of women are able to access facilities for at least one ANC visit^64^.

In conclusion, we found very high rates of unintended pregnancy and very low modern FP use in the context of a resource-limited, high burden setting of poor MCH in rural PNG. The combination of low levels of knowledge, poor uptake, and very limited contraceptive choice highlights the urgent need for gender-inclusive, context-specific supply- and demand-side FP interventions at individual, family and community levels.

## Data Availability

The clinical and epidemiologic datasets generated and analysed during the current study are not publicly available due to ethics considerations, but further data may be provided from the authors upon reasonable request.
